# The Skin and Intestinal Microbiota and Their Specific Innate Immune Systems

**DOI:** 10.3389/fimmu.2019.02950

**Published:** 2019-12-17

**Authors:** Margaret Coates, Min Jin Lee, Diana Norton, Amanda S. MacLeod

**Affiliations:** ^1^Department of Dermatology, Duke University, Durham, NC, United States; ^2^Department of Molecular Genetics and Microbiology, Duke University, Durham, NC, United States; ^3^Department of Immunology, Duke University, Durham, NC, United States; ^4^Pinnell Center for Investigative Dermatology, Duke University, Durham, NC, United States

**Keywords:** skin, intestine, microbiome, innate immunity, AMPs

## Abstract

The skin and intestine are active organs of the immune system that are constantly exposed to the outside environment. They support diverse microbiota, both commensal and pathogenic, which encompass bacteria, viruses, fungi, and parasites. The skin and intestine must maintain homeostasis with the diversity of commensal organisms present on epithelial surfaces. Here we review the current literature pertaining to epithelial barrier formation, microbial composition, and the complex regulatory mechanisms governing the interaction between the innate immune system and microbiota in the skin and intestine. We also compare and contrast the skin and intestine—two different organ systems responsible creating a protective barrier against the external environment, each of which has unique mechanisms for interaction with commensal populations and host repair.

## Introduction

The skin and intestine both rely on multifaceted mechanisms to maintain homeostasis and protect against invading microbes. Components essential for proper homeostasis between the external environments and the skin or intestine include the physical barrier formed by epithelial cells, the chemical barrier, the presence of beneficial commensal microbiota, and finally the tissue-resident and infiltrating immune cells. The barrier surfaces of the skin and intestine are not only habitats for commensal microbiota, but they also represent potential entry sites for pathogens, including bacteria, viruses, fungi, and parasites. The direct interface between the epithelial tissue barrier and microbiota poses a challenge for the barrier-lining epithelial cells and resident immune cells to distinguish dangerous pathogens from commensals and respond accordingly. Therefore, complex regulatory mechanisms have evolved to allow for delicate coordination between host tissues and their resident microbes. In this review, we provide an overview of the epithelial anatomy of the skin and intestine and interactions between host and microbiota at these surfaces. We focus on the role of microbiota and the innate immune system at homeostasis, in protection against infections, and in tissue repair of the skin and intestine.

## Structure of the Protective Barrier

The large surface areas of the skin and intestine—at least 30 m^2^ of skin in adults and about 400 m^2^ of intestinal epithelium—provides an expansive interface for interaction with the outside environment and increases the risk of invasion by pathogens (1–3). Given their extensive surface areas, the skin and intestine not only harbor millions of commensal microbiota, but they also must rely on multiple protective strategies to prevent entry of pathogens. As a result, the skin and intestine have developed site-specific physical, chemical, microbial, and immunologic barriers to maintain health and eradicate pathogenic bacteria.

### Physical Barrier

**Figure 1:**
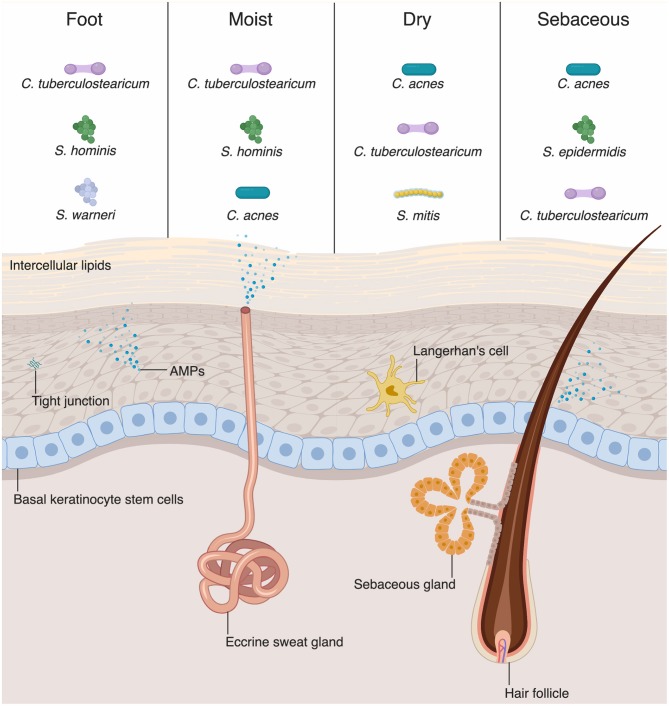
Skin-microbial interactions promote innate immune function. The skin is an active immune organ whose function is augmented by the presence of commensal microbiota. The epidermis is made up of numerous keratinocytes. The stratum corneum is sealed via intracellular lipids, and other epidermal keratinocytes are connected via tight junctions. Dermal appendages include sweat glands, hair follicles, and sebaceous glands, all of which contribute to immune function. Keratinocytes and dermal appendages release antimicrobial peptides and proteins (AMPs), which provide defense against pathogenic microbes. A number of bacteria species are commensal colonizers of the skin surface. The top three bacterial species for each skin site are shown (4). Dry and sebaceous sites are colonized predominantly by *Cutibacterium acnes*, whereas moist sites and the foot are colonized chiefly by *Corynebacterium tuberculostearicum*.

The physical barrier of the skin and intestine provides the first line of defense against external perturbation at these sites. The physical barrier of the skin is formed by numerous layers of epidermal and dermal keratinocytes (Figure 1). The outermost layer of the epidermis is the stratum corneum, composed of as many as 100 layers of keratinized cell envelopes (corneocytes) that form a protective barrier (5). Barrier lipids, derived from lamellar bodies form an occlusive matrix between corneocytes (6). Deeper epidermal layers, including the stratum granulosum and stratum spinosum, are major producers of keratin filaments, which form a structural support for the epidermis (5). Finally, the basal layer of the epidermis contains stem cells that proliferate in homeostatic conditions and in response to injury in order to reconstitute the physical epidermal barrier. Epidermal keratinocytes maintain tight physical contact through tight junctions and adherens junctions, which form protective layer that is nearly impermeable to microbes. In addition to providing physical protection at the skin barrier, tight junction proteins, such as zona occludins proteins, play roles in proliferation and differentiation of keratinocytes in the skin, allowing re-establishment of the barrier against microbes after breach of the skin from wounding (7).

**Figure 2:**
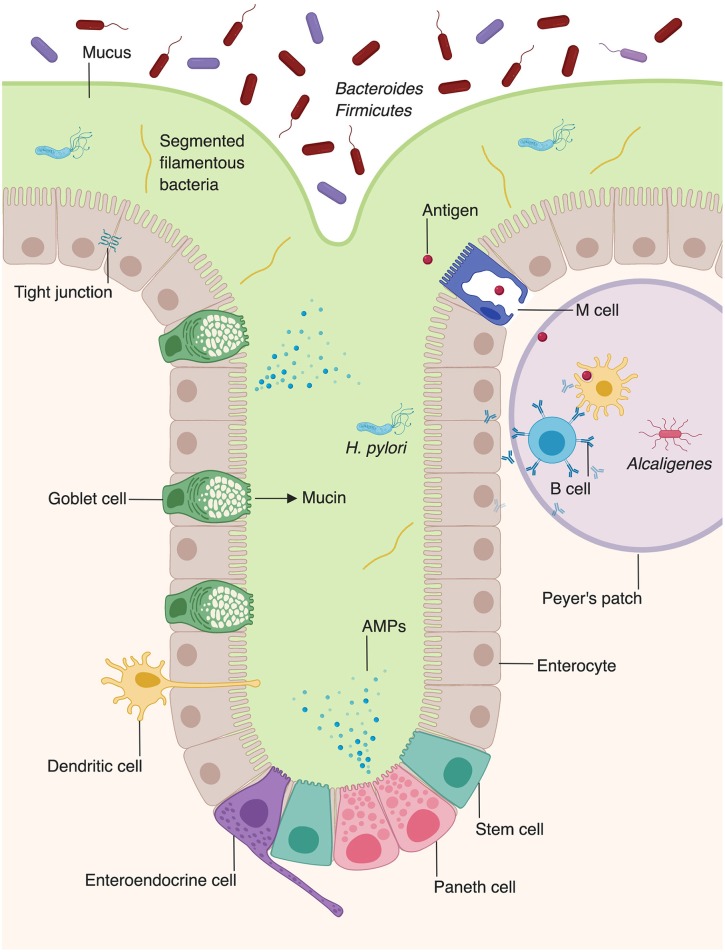
Microbiota augment intestinal innate immunity. Intestinal epithelial cells, which make up the physical barrier of the intestine, secrete antimicrobial peptides and proteins (AMPs). Goblet cells secrete mucus which forms an additional layer of protection against pathogens. Dendritic cells present antigen to B cells within Peyer's patches, stimulating them to secrete IgA. The intestine provides unique niches in which commensal bacteria thrive. *Bacteroides* and *Firmicutes* species comprise the majority of luminal bacteria, whereas segmented filamentous bacteria and *Helicobacter pylori* can penetrate into the mucus layer of the intestine (8, 9). *Alcaligenes* species are able to inhabit Peyer's patches (10).

In contrast to the stratified squamous epithelium of the skin, the intestinal barrier is composed of a single layer of columnar epithelial cells (Figure 2) (11). However, this single layer of intestinal epithelial cells (IECs) is made of diverse cell types with absorptive, secretory, and immune function (2). This includes not only the absorptive enterocytes, which encompass the majority of IECs, but also secretory goblet cells, Paneth cells, and enteroendocrine cells. All cells that make up the intestinal barrier are constantly renewed by intestinal epithelial stem cells located in the bases of mucosal crypts (Figure 2). As in the skin, IECs are connected via tight junctions, which form a strong physical barrier that impedes translocation of pathogenic microbes or toxins.

### Chemical Barrier

The chemical barrier of the skin is formed by numerous secreted lipids and acids. As previously mentioned, the lipid layer secreted by lamellar bodies, is important for maintaining an occlusive matrix between cells and among layers of the stratum corneum (12, 13). Site-specific lipid content also influences the microbial composition of various cutaneous body sites (4, 14). In fact, microbial composition is relatively homogenous among multiple sebaceous sites but varies greatly between sebaceous and dry skin sites (4). Pathogenic microbes are also directly inhibited by some lipids or free fatty acids. For example, sapienic acid can efficiently inhibit pathogenic *Staphylococcus aureus* (*S. aureus*), but does not have sufficient activity against *Staphylococcus epidermidis* (*S. epidermidis*) (15). Overall, the chemical barrier formed by epidermal lipids and fatty acids is important for modulating microbial survival at the skin surface.

In addition, the stratum corneum of the epidermis maintains an acidic pH under homeostatic conditions. The term “acid mantle” has been used to describe the acidic condition of the stratum corneum (16). This acidic pH is important for skin barrier function and microbial defense by providing hostile environment for certain microorganisms (12). Furthermore, there are a number of pH-dependent enzymes that are critical for synthesis, production and maintenance of the lipid composition in the skin. Lipids, such as triglycerides and cholesterol, are hydrolyzed by skin-resident bacteria and yeasts into free fatty acids. Free fatty acids maintain a low pH that inhibits growth of pathogenic species such as *Staphylococcus aureus* (*S. aureus*), while allowing persistence of commensal bacteria such as coagulase negative *Staphylococcus* and *Corynebacterium* (1, 17).

The intestine relies on goblet cells to secrete a thick layer of jelly-like mucus made of glycoproteins to separate luminal bacteria from epithelial cells and create a distinct protected zone (Figure 2) (18). Mucins create both a chemical and a physical barrier between the intestinal lumen and EICs, and can even directly modulate expression of tolerogenic and inflammatory cytokines (19). In addition to providing physical protection, mucin layer is also rich in secretory IgA and antimicrobial proteins (AMPs) that provide a chemical immune defense against potential invading microorganisms (20, 21). Mucin synthesis is increased by short chain fatty acids (SCFAs), a fermentation product of bacterial metabolism (22). Furthermore, mucin production is decreased in germ-free mice, but production of mucin can be rescued by activation of microbe-sensing receptors, suggesting that commensal microbes enhance the intestinal barrier (2, 23). The composition of the mucin layer differs between the small and large intestine. The mucous layer of the small intestine is physically penetrable by bacteria, and epithelial cells are protected via secreted AMPs (24). In contrast, the large intestine contains both penetrable outer mucus layer and an impenetrable inner mucous layer (25).

## Diversity of Commensal Microbiota

With the rise of new techniques such as 16S and whole genome metagenomic shotgun sequencing, we have begun to understand in greater detail the diversity and functions of microbiota that colonize the skin and intestine (14, 26). The skin and intestine support a tremendous diversity and number of microbiota. In both the skin and intestine, commensal microbiota are important for maintaining epithelial homeostasis and overall health of the tissue (4, 27).

### Site-Specific Differential Composition of Microbiota

Although differing profoundly in taxonomic composition, the skin and intestine are similar in that the microbial composition varies among sites and niches. Recent sequencing studies have extensively mapped the species inhabiting various skin or body sites with different compositions, including wet, dry, and sebaceous sites (Figure 1) (14, 28). Distinct skin sites contain unique distribution of bacteria, in part governed by the lipid composition of a skin site (14). For example, sebaceous gland-rich areas, such as the glabella and back, are colonized most predominantly by *Cutibacterium* (formerly known as *Propionibacterium*) species, which are closely associated with the common condition acne vulgaris (14). Moist sites, such as the axilla and interdigital web spaces, are largely colonized by *Corynebacteria* and *Staphylococci* species (14).

In addition to bacteria, which are the most abundant kingdoms of organisms found on the skin, numerous fungi and viruses inhabit the skin (14). In contrast to bacteria, which are found in nearly all bodies sites and whose composition is governed by physiologic conditions, fungal distribution varies based on distinct body sites rather than physiologic conditions (29). The core body and arms have a relatively homogenous fungal composition and are predominantly colonized by *Malassezia* species, whereas the foot harbors a much greater fungal diversity (29). Viral composition, predominantly *Polyomaviridae* and *Papillomaviridae*, shows most diversity between individuals, rather than depending on body site or composition (28).

In contrast to the skin, which is inhabited by aerobic bacteria, aerotolerant anaerobes, or facultative anaerobes, the intestine is mostly colonized by anaerobes, such as bacteria of the phyla *Bacteroidetes* and *Firmicutes* (Figure 2) (8, 14). Whereas, the microbial composition of the skin is largely determined by environmental factors such as the presence or absence of sebum, the intestinal microbiota is dependent on location, niche, and external factors, such as diet (14, 30). The large intestine harbors a higher microbial diversity and density within individuals than the small intestine (31, 32). However, evidence suggests that the microbial composition of the small intestine is more dynamic than that of the large intestine, with large temporal fluctuations in ileal microbial constituents within a single day (33). Fewer studies have examined the microbial composition of the small intestine, compared to the large intestine. However, one study utilized 16s rRNA sequencing to examine the bacterial compositions of the jejunum, ileum, cecum, and recto-sigmoid colon (32). Facultative anaerobic bacteria were present in all four locations along the gastrointestinal tract. Lactobacilli, streptococci, and *Enterococcus* were detected at high frequencies in the jejunum and ileum. In addition to facultative anaerobes, which were the major operational taxonomic unit in both the small and large intestine, the large intestine was also found to contain obligate anaerobic bacteria (32).

Within the small or large intestine, the environmental niches can be luminal, mucus-associated, epithelial-associated, or lymphoid tissue-resident (30). Which phyla of bacteria inhabit a specific intestinal niche depends significantly on the characteristics of both the bacteria and the niche itself. Luminal bacteria are largely of the *Bacteroidetes* and *Firmicutes* phyla, and represent the largest percent of intestinal biomass (8). Recent studies have illuminated that the outer mucus layer of the large intestine forms a unique “mucus-associated” microbial niche with distinct bacterial communities (9). Specifically, bacteria of this niche are adapted to thrive in high levels of bioavailable iron and carbon, an ability conferred by their distinct genome-encoded metabolic and mucolytic activities. For example, *Helicobacter pylori* secretes urease to increase the pH of the mucin layer and disrupts the strong glycoprotein bonds, which allows it to burrow into the stomach mucosa (34).

The epithelial-associated bacteria make up a smaller proportion of intestine bacteria since fewer bacteria are able to infiltrate through the mucous layer (30). Epithelial-associated bacteria express distinct genes that allow them to colonize epithelial cells. For example, expression of commensal colonization factor (*Ccf* ) genes allows *Bacillus fragilis* to metabolize carbohydrates present in the intestinal lumen and therefore promotes their colonization of intestinal epithelium, illustrating the importance of nutrient-specific factors in determining microbial composition (35). Furthermore, although *B. fragilis* is an anaerobic bacteria and thrives predominantly in the intestinal lumen, it also grows well in nanomolar oxygen concentrations, such as that found in intestinal crypts (36). Epithelial-associated bacteria are also important for proper function of the intestinal immune system. For example, segmented filamentous bacteria adhere tightly to EICs and induces a Th17 response, conferring protection against pathogenic mucosal bacteria (30). Intestine-associated lymphoid tissues, including Peyer's patches and lymphoid follicles, are colonized largely by *Alcaligenes* species (10). However, it should be noted that, under homeostatic conditions, these bacteria do not spread to the spleen or produce a systemic IgG response. Colonization of intestine-associated lymphoid tissues by these bacteria only results in the local production of *Alcaligenes*-specific IgA antibodies, highlighting the tolerogenic response to a lymphoid tissue-resident bacteria (10). Overall, the special distribution of intestinal bacteria is dependent on niche-specific factors, such as availability of nutrients or site specific microbial-host interactions.

### Temporal Changes in the Commensal Microbiome

Commensal species, which can vary according to topography and anatomic environments, also undergo temporal changes as humans develop over time. It was previously thought that *in utero* fetuses were in a germ-free environment. However, data have shown that bacteria can be cultured from the umbilical cord and meconium of healthy full term babies (37, 38). 16S rRNA gene sequencing recently confirmed the presence of microbiota in newborn meconium and amniotic fluid (39). Meconium samples contained bacterial DNA, the majority of which mapped to *Pelomonas puraquae*. Conversely, amniotic fluid bacterial DNA mapped to skin commensal species such as *Cutibacterium acnes* and *Staphylococcus* species (39). The neonatal skin is first colonized by microbes present in the birth canal. Subsequently, an infant's microbiome is shaped by contact with the outside environment. Studies have shown that the skin flora of a baby is largely shaped by the mother's microbiome at birth and that there are notable differences in both skin and intestinal microbiota between infants born naturally or by C-section (40). The infant can also be exposed to viruses, such as herpes simplex virus type 2, present in the mother's vaginal tract (41). Over the course of the first year of life, the infant's skin microbiome is established and begins to resemble that of adults (42).

The intestine similarly has a temporal shift in its microbial flora as the baby transitions from an exclusively milk diet to solid foods (42). An initial diet of breast milk results in high levels of facultative and obligate anaerobes, such as *Escherichia coli, Streptococcus*, and *Bifidobacterium* species (43). Breast milk provides a source of human milk oligosaccharides and milk glycoconjugates, which are consumed by *Bifidobacterium* species (44). *Bacteroides* and *Clostridia* species predominate as babies are weaned and ingest more complex carbohydrates (43). *Clostridia* species are particularly specialized in degrading plant polysaccharides and are therefore able to thrive in the intestine once complex carbohydrates are introduced into the infant diet (45).

Beyond the early years of life, both skin and intestinal microbiome become more stable, and within-individual variation in microbial communities over time is much less than between-individual variations (28, 46, 47). Despite the relative stability of the skin microbiome, it is less stable over time than the intestinal microbiome (48). Furthermore, the level of microbial stability over time is significantly different among individuals; some individuals have a very stable skin microbiome, whereas others do not. Skin sites that have extensive environmental contact, such as the palm, display the least stability in microbial composition. Interestingly, individuals with a more diverse intestinal microbiome (in terms of number of bacteria species) also have a more stable microbiome over time, whereas individuals with a more diverse skin microbiome have a less stable microbiome over time (48). Microbial diversity decreases in the elderly, coinciding with a decline in immunocompetence in older populations (49). The complex shifts in establishing a commensal population depending on skin and intestinal sites, physiologic conditions, and temporality highlight the importance of finely-tuned interactions between host and microbiota.

### Environmental Influences on Microbiome Composition

In both the skin and intestine, microbial diversity is influenced by a plethora of exogenous factors, including diet, antibiotic use, and obesity (50–52). In the skin, treatment with topical or systemic antibiotics has been linked to shifts in the cutaneous microbiome. For example, use of topical antibiotics, such a bacitracin, neomycin, and polymyxin B (found in the commonly-used triple antibiotic ointment) lead to decreased commensal *Staphylococcus* strain in mice (53). Oral isotretinoin or tetracycline treatment leads to decreased abundance of *Cutibacterium* on the skin and the microbiome of sebaceous areas shifts to mimic that of dry sites, containing a greater proportion of *Staphylococcus* and *Streptococcus* species (54).

Diet is a strong driver of microbial composition in the intestine. An animal-based diet increases the abundance of bacteria that are bile-tolerant, such as *Alistipes, Bilophila*, and *Bacteroides* (50). In contrast, a vegan or vegetarian diet is associated with an increased prevalence of lactic acid bacteria, including *Ruminococcus, Eubacterium rectale, and Roseburia* (55). *Prevotella* species predominate in humans whose diets are high in carbohydrates and simple sugars (56). High fiber diets lead to a higher abundance of bacteria that ferment fiber into SCFAs, which have a broad range of beneficial effects, including immunomodulatory properties (57). Diet can even influence the circadian dynamics of intestinal microbiota: diet-induced obesity causes a dampening of diurnal variations in microbial composition (58).

## Maintaining Host-Commensal Homeostasis Via Innate Immunity

The skin and intestine have developed symbiotic relationships with commensal microbes and established a homeostasis that balances tolerating commensal microbes while defending against pathogens.

### Commensal Microbiota Help Maintain Homeostasis in the Skin and Intestine

In the skin, the presence of commensal bacteria is crucial for maintenance of a healthy cutaneous environment. In development, skin immune tolerance begins developing in the post-natal period when T reg cells begin expressing the pathogen-specific FOXP3 transcription factor, coinciding with commensal colonization (59). Later in development, the continued presence of skin commensal bacteria modulates production of numerous cytokines and AMPs that help to protect the skin against pathogens. For example, commensal bacteria such as *S. epidermidis* can induce production of various cytokines by IL-17^+^CD8^+^ T cells (60). *S. epidermidis* can also produce ligands that suppress inappropriate immune activation by inhibiting production of tumor necrosis factor-α and IL-6 (61). Recent work demonstrated that germ-free mice have decreased expression of Toll-like receptors (TLRs), AMPs, complement cascades, and IL-1 cytokine signaling in the skin, when compared to specific pathogen free mice (62).

It is also well-established that bacterial colonization is essential for maturation of the intestinal innate immune system and, as in the skin, commensal microbiota work in tandem with the immune system to protect the host against pathogens (63, 64). For example, *Bacteroides fragilis* (*B. fragilis*) and commensal *Clostridium* cluster such as IV and XIVa can accumulate Foxp3^+^ T_reg_ cells in mice and help build immune tolerance to the commensal microbiome (65, 66). In addition, the lamina propria harbors macrophages, whose function is phagocytosis of pathogens. However, lamina propria-associated macrophages do not express as strong proinflammatory phagocytic responses as macrophages at other sites (67). This suggests adaptation of the host in response to a large population of commensal microbiota, which minimizes unnecessary inflammation (34).

### Innate Immune Responses Upon Barrier Injury Are Modulated by Commensal Microbiota

The physical and chemical barriers discussed above are crucial in preventing tissue penetration of the microbes by decreasing direct contact between them. However, pathogenic microbes may gain access to the tissue when there is a breach of these barriers. Disruption of the skin barrier may occur through physical cut or toxic chemical exposure. Barrier disruption is also associated with chronic intestinal diseases such as inflammatory bowel disease, obesity, and diabetes, all of which can increase the intestinal permeability (68). In the following section, we discuss innate immune mechanisms upon barrier breach and how they are modulated by commensal bacteria.

Prompt recognition and eradication of pathogens are necessary to prevent infection. The innate immune system provides a first line of defense against pathogens upon barrier breach. Recent evidence has illuminated the role of commensal microbes in strengthening innate immune defense against pathogens (69). Pattern recognition receptors (PRRs) interact with microbe- or pathogen-associated molecular patterns (MAMPs or PAMPs) such as lipopolysaccharide (LPS) and peptidoglycan (PG) on bacteria and nucleic acids from bacteria (70). Upon activation of PRRs, downstream signaling leads to release of inflammatory cytokines or activation of immune cells. In the skin, PRRs are present on immune cells and keratinocytes. TLR stimulation can mediate direct antimicrobial action through the stimulation of macrophages to undergo phagocytosis and can also induce cytokines that mediate the differentiation of monocytes into macrophages and dendritic cells (70, 71). Commensal microbes secrete molecules which may act directly as TLR ligands; *S. epidermidis* secretes multiple small molecules that act as TLR2 and EGFR agonists, stimulating production of AMPs that have activity against group A *Streptococcus* and *S. aureus* (72–74).

AMPs play a critical role in innate immunity by acting under homeostatic conditions and destroying pathogenic microbes through various mechanisms (75). Keratinocytes, the major cell type in the skin, produce various AMPs (Figure 1) (49, 76). Human β-defensin-1 (hBD-1) is constitutively expressed by keratinocytes whereas hBD-2 and −3 are upregulated in response to inflammation (77–79). Human cathelicidin (hCAP-18) is cleaved and processed to the active form of antimicrobial peptide, LL-37 which then disrupts microbial membranes (80). Some specialized keratinocytes that make up appendages such as hair follicles, sweat glands, and sebaceous glands have various AMPs pertinent to their microenvironments (Figure 1). For example, dermcidin is traditionally thought to be a sweat-gland specific AMP, yet there are emerging evidences that it is also produced by sebaceous gland in humans and mice (81, 82). Sebaceous glands also produce cathelicidin and hBD-2 (83, 84). Commensal bacteria have been shown to secrete AMPs. *S. epidermidis* secretes phenol-soluble modulin γ and δ that have antibiotic effects on *S. aureus* (85). Commensal bacteria can also act on lipids secreted from sebaceous glands and hydrolyze them to free fatty acids (FFAs) (86). FFAs have intrinsic antibacterial effects against various Gram-positive bacteria; sapienic acid has activity against methicillin-resistant *S. aureus* (MRSA) (87). Furthermore, FFAs can induce sebocytes to upregulate expression of hBD-2 (88).

Just as external environmental effects are known to modulate skin microbial composition, environmental factors also regulate microbial recognition and AMP production in the skin. Ligand-dependent activation of the vitamin D receptor (VDR) is required for recruitment of macrophages to the injury site after wounding (89, 90). Genes coding for TLR2 are induced by the presence of 1,25-dihydroxy vitamin D3, which is regulated in part by exposure to UV light (83). Furthermore, vitamin D3-induced expression of TLR2 leads to cathelicidin production upon exposure to microbial components. Conversely, TLR2 activation can lead to increased expression of the VDR, which can be activated by vitamin D3 to produce cathelicidin (91). Vitamin D3-eluting wound nanodressings have even been shown to increased cathelicidin expression in human skin wounded explants (92).

Similarly to epidermal keratinocytes, IECs express PRRs, such as TLRS, NOD-like receptors (NLRs), and RIG-I-like receptors (RLRs) (93). PRR signaling is important both in homeostasis and in response to pathogenic bacteria, highlighting the diverse functions of innate immunity at steady-state and under disease conditions (94–96). PRRs also respond differently under homeostatic vs. inflammatory conditions, in part because of the presence of damage-associated molecular patterns (DAMPs), which are released by injured epithelial cells (97). Interestingly, steady-state activation of TLRs by commensal intestinal microbiota is also important for proper intestinal homeostasis. For example, mice deficient in TLRs, downstream signaling components of the TLR pathway, or normal commensal microbiota all displayed greater morbidity and mortality following intestinal epithelial disruption (95). Furthermore, TLR activation by commensal bacteria can enhance the protective function of tight junctions against pathogens by strengthening the zonula occludens-1 protein (94). The activated macrophages also signal repair pathway that promotes rapid enterocyte proliferation to repair the tissue defect by producing growth factors (34, 43). This highlights the importance of synergistic activity of commensal microbiota and host innate immunity in maintenance of a healthy epithelium.

In addition to producing barrier-protective mucins, EICs are also a rich source of AMPs (Figure 2) (2). Enterocytes produce AMPs including regenerating islet-derived protein III_γ_ (REGIII_γ_) and numerous β-defensins, which play diverse antimicrobial roles, including spatial segregation of bacteria (98, 99). Beyond their roles in barrier formation and production of AMPs, enterocytes also facilitate the translocation of secretory immunoglobulins, particularly IgA, across the intestinal wall (100). Paneth cells, present in intestinal crypts, produce additional AMPs, including α-defensins, lysozyme, and phospholipase A2 (98, 101, 102).

Intestinal commensals are able to induce AMP production in the intestine. *Lactobacillus* and probiotic *E. coli* strains are able to induce secretion of hBD-2 from enterocytes (103, 104). Commensal microbiota in the intestine are also capable of producing molecules that protect the host from chronic inflammatory diseases. For example, polysaccharide A, produced by *B. fragilis*, prevents inflammatory bowel disease (IBD) via an IL-10-producing CD4^+^ T cell-dependent mechanism (105). SCFAs produced by commensals of the genuses *Bifidobacterium* and *Bacteroides* interact with the G-protein-coupled receptor 43 (GPCR43) (106). Mice deficient in GPCR43 have impaired resolution of inflammation in models of IBD, arthritis and asthma. Similarly to vitamin D3-dependent regulation of AMPs in the skin, butyrate regulates AMP production in the intestine. Butyrate is a SCFA that is produced by fermentation of carbohydrates in the lumen by intestinal bacteria (107). Butyrate strongly induces cathelicidin production in colonic epithelial cells, and moderately induces h-BD1 and h-BD2 (108). Factors produced by commensal bacteria in the intestine may also prevent injury to IECs or facilitate intestinal repair. Numerous commensal bacteria produce compounds that prevent damage by noxious stimuli. Competence and sporulation factor (CSF) produced by *Bacillus subtilis* activates the mitogen-activated protein kinase (MAPK) pathway to protect epithelial cells from oxidative stress (109). Similarly, *Lactobacillus rhamnosus* produces two compounds, p75 and p40, which prevent cytokine-induced apoptosis of IECs through epidermal growth factor receptor (EGFR) signaling, which activates anti-apoptotic Akt/protein kinase B (110–112). Tight junction assembly is promoted by *Bifidobacterium* and butyrate from various bacteria, underscoring the ability of commensal bacteria to promote intestinal barrier function (113–115).

## Conclusion

The skin and intestine are both active immune organs that are under constant environmental exposure. Therefore, complex regulatory mechanisms have evolved to maintain homeostasis. In addition to acting as physical barriers, epithelial cells of the skin and intestine produce AMPs, which act as endogenous antibiotics to protect against potential pathogens. Immune cells also constantly surveil both of these surfaces. More recently, it has been appreciated commensal microbiota may induce beneficial, tolerogenic immune responses under homeostasis or prime the immune system to fight against pathogens upon barrier breach. Some commensal bacteria may even produce AMPs on their own. An improved understanding of beneficial microbial-immune interactions has paved the way for new research involving exogenous supplementation of skin and intestinal microbial populations. For example, topical application of Gram-negative bacterial species obtained from healthy human volunteers improved atopic dermatitis in a mouse model (116). Manipulation of intestinal microbiota may be a promising therapeutic option for the treatment of numerous disease, including obesity, IBD, colorectal cancer, and liver disease (117). Although further studies will be needed to validate the safety and efficacy of a microbial-based therapeutic approach, it is clear that a healthy skin and intestinal microbiome is crucial for healthy epithelial homeostasis and immunity.

## Author Contributions

MC, ML, DN, and AM contributed to conception and design of the review. MC, ML, and DN performed initial literature search and wrote the first draft of the manuscript. All authors contributed to manuscript revision, read and approved the submitted version. AM supervised all aspects of the review and manuscript writing and is the corresponding author.

### Conflict of Interest

The authors declare that the research was conducted in the absence of any commercial or financial relationships that could be construed as a potential conflict of interest.
